# Quantifying Methionine Requirements for Two Strains of Juvenile Nile Tilapia (*Oreochromis niloticus*) Fed Diets Containing Black Soldier Fly Larvae Meal

**DOI:** 10.1155/anu/9270315

**Published:** 2026-11-15

**Authors:** Jimmy B. Mboya, Menaga Meenakshisundaram, Erick Ogello, Jonathan Munguti, Kartik Baruah, Ahmad Fatan Nurulhuda, Matthew A. G. Owen, Rodrigue Yossa, Chrysantus M. Tanga

**Affiliations:** ^1^ International Centre of Insect Physiology and Ecology (icipe) Nairobi Kenya icipe.org; ^2^ Department of Animal and Fisheries Sciences Maseno University Maseno Kenya maseno.ac.ke; ^3^ Kenya Marine and Fisheries Research Institute (KMFRI) Sagana Kenya kmfri.co.ke; ^4^ Department of Applied Animal Science and Welfare Faculty of Veterinary Medicine and Animal Sciences Swedish University of Agricultural Sciences Uppsala Sweden slu.se; ^5^ WorldFish Jalan Batu Maung, Batu Maung Bayan Lepas Malaysia worldfishcenter.org; ^6^ CGIAR Sustainable Animal and Aquatic Foods (SAAF) Program Nairobi Kenya

**Keywords:** alternative fish feed, black soldier fly larvae protein, circular bioeconomy, growth performance, nutrient requirements

## Abstract

This study investigated the optimal dietary methionine requirements of local and genetically improved strains of Nile tilapia (*Oreochromis niloticus*) fed diets with constant inclusion levels of black soldier fly larvae meal (BSFL). Six isonitrogenous (∿35% crude protein [CP]), isolipidic (∿11% crude fat) and isoenergetic (∿492 kcal gross energy/100 g) diets (D0.36, D0.96, D1.05, D1.15, D1.23 and D1.41) were formulated with graded levels of DL‐methionine (0%, 0.67%, 0.77%, 0.87%, 0.97% and 1.17%, respectively) at the expense of carboxymethyl cellulose to give analysed methionine levels of 3.6, 9.6, 10.5, 11.5, 12.3 and 14.1 g/kg of diet, with a constant cysteine level of 4.2 g/kg. Thirty‐five fish of each strain (4.08 ± 0.12 g) were randomly stocked in each 100 L plastic tank, with three replicate tanks per treatment, in a recirculating aquaculture system (RAS) and fed to apparent satiation twice daily for 84 days. There were significant effects of dietary methionine, strain and their interaction on all analysed parameters (*p* < 0.05), except survival rate and albumin levels. Significantly higher growth performance, feed utilisation, haemato‐biochemical and antioxidant profiles were recorded with a methionine inclusion of 10.5 g/kg in the diet for the local strain and 11.5 g/kg in the diet for the improved strain (*p* < 0.05). Quadratic regression estimated optimum dietary methionine requirements at 9.08–9.64 g/kg of the diet for the local strain and at 9.13–9.55 g/kg of the diet for the improved strain, with a cysteine level of 4.2 g/kg. These findings suggest that aligning dietary formulations with strain‐specific nutritional requirements is crucial for optimal fish performance, successful fish farming and sustainable aquaculture.

## 1. Introduction

Nile tilapia (*Oreochromis niloticus*) is a widely cultivated fish species that serves as an accessible and cost‐effective protein source for consumers [1, 2]. As an omnivorous species, tilapia ranks among the most extensively farmed fish worldwide and is currently the second most produced aquaculture species globally after carps due to its high consumer demand and the rapid expansion of the aquaculture sector [3, 4]. Importantly, the commercial culture of the fish depends greatly on its diet. Studies have indicated that insect‐derived protein meals may offer a more sustainable substitute for traditional protein sources used in aquaculture [5–7]. Black soldier fly (*Hermetia illucens*) larvae meal (BSFL), when used in place of fishmeal, has demonstrated favourable results in *O. niloticus* when inclusion levels are optimised, with several studies recommending 40%–75% fishmeal replacement in diets of fish [5, 8–10]. However, BSFL‐containing diets pose nutritional limitations as BSFL is low in essential sulphur‐containing amino acids, especially methionine, compared with fishmeal [11, 12].

Methionine is an essential sulphur‐containing amino acid and a critical component of fish nutrition, playing a pivotal role in protein synthesis, growth, immune function and overall metabolic health in aquaculture species [13–15]. Methionine also modulates antioxidant enzyme activity and biochemical and haematological markers in fish, thereby improving immune function and stress tolerance [16, 17]. The dietary methionine requirement for *O. niloticus* has been reported by previous studies. For instance, Xiang et al. [18] reported 11.3–11.6 g/kg of diet (with cysteine level at 3.0 g/kg of diet), He et al. [19] reported 9.12 g/kg of diet (with a cysteine level of 0.83 g/kg of diet), Garg et al. [20] reported 12.1–14.1 g/kg of diet (with a cysteine level of 3.2 g/kg of diet) and Yossa et al. [21] reported 7.5–8.0 g/kg of diet (with cysteine level of 5.0 g/kg of diet).

In addition to dietary considerations, genetic variations in fish can influence their dietary methionine requirements. Improved *O. niloticus* strains have been selectively bred for enhanced growth and metabolism [22–25]. Previous research underscores the importance of understanding inter‐strain differences to develop tailored feeding strategies that meet strain‐specific nutritional requirements to optimise biological outcomes in diverse aquaculture settings [26, 27].

Despite the growing interest in methionine inclusion in fish diets, there is limited empirical data on the optimal dietary methionine levels in insect‐containing feeds. Moreover, comparative evaluations of methionine requirements in local and improved *O. niloticus* strains for BSFL‐containing diets are lacking. These gaps hinder the development of optimised, strain‐specific feed formulations for sustainable farming of *O. niloticus*. Therefore, this study investigated the growth performance, feed utilisation, somatic, biochemical, antioxidant and haematological indices of local and improved *O. niloticus* strains available in Kenya fed diets containing graded levels of dietary methionine in BSFL‐containing diets. The findings will contribute to a deeper understanding of the amino acid metabolism and the development of strain‐specific sustainable feeds to enhance aquaculture production.

## 2. Materials and Methods

### 2.1. Ethical Considerations

All experimental procedures involving fish were performed following the Animal Research: Reporting of In Vivo Experiments (ARRIVE) Guidelines, with approval from the National Commission for Science, Technology, and Innovation (NACOSTI), with research permit number NACOSTI/P/24/40962. Fish were frequently monitored to ensure that they were in good condition throughout the experimental period.

### 2.2. Study Site, Experimental Design and Operation

The study was conducted at the International Centre of Insect Physiology and Ecology (*icipe*), Duduville Campus in Nairobi, Kenya. A completely randomised 2 × 6 factorial design with three replicates was used in the experiment. The two factors were (1) graded levels of methionine and (2) two strains of *O. niloticus*. This comprised 12 treatments, each replicated three times, for a total of 36 experimental units. Thirty‐six (36) tanks were used, each measuring 68 cm in length × 38 cm in width × 40 cm in height, with a water volume of 100 L each, installed in a recirculating aquaculture system (RAS). Individual air stones connected to a blower were used to ensure aeration in each tank to maintain a dissolved oxygen (DO) level of 5–7 mg/L. The system was housed in a room illuminated with sunlight, continuously providing 12 h of light per day.

### 2.3. Experimental Diets

Defatted BSFL was used in an equal ratio with fish meal from silver cyprinid (*Rastrineobola argentea*) as the primary sources of crude protein (CP) in the diets, in addition to other ingredients, to formulate six isonitrogenous, isolipidic and isoenergetic diets (Table 1). Cassava flour served as a starch source to contribute to the binding and physical properties of the extruded diets. The diets were formulated by gradually replacing carboxymethyl cellulose (henceforth referred to as cellulose in this paper) with feed‐grade DL‐methionine at 0%, 0.67%, 0.77%, 0.87%, 0.97% and 1.17% [21, 28]. The defatted BSFL was obtained from Sanergy Limited Company, Kenya; ascorbic acid (Tiger C from Anhui Tiger Biotech Ltd Company, Anhui, China) was obtained from Arichem Ltd Company, Kenya, while all the other ingredients, including DL‐methionine (Rhodimet, Adisseo, France) and L‐lysine hydrochloride (HCl; ≥98.5% purity; Polifar Group Limited), were obtained from certified feed millers in Thika Industrial Park, Kenya.

**Table 1:** Diet formulation and proximate composition of the experimental diets fed to the local and improved Nile tilapia strains for 84 days.

Ingredients (%)	Experimental diets					
	D0.36	D0.96	D1.05	D1.15	D1.23	D1.41
DL‐methionine	0.00	0.67	0.77	0.87	0.97	1.17
Carboxymethyl cellulose	1.20	0.53	0.43	0.33	0.23	0.03
BSFL	22.00	22.00	22.00	22.00	22.00	22.00
Fish meal	22.00	22.00	22.00	22.00	22.00	22.00
Groundnut cake	32.50	32.50	32.50	32.50	32.50	32.50
Wheat bran	3.24	3.24	3.24	3.24	3.24	3.24
Cassava flour	13.80	13.80	13.80	13.80	13.80	13.80
Soybean oil	2.00	2.00	2.00	2.00	2.00	2.00
Bone meal	2.00	2.00	2.00	2.00	2.00	2.00
Premix	0.50	0.50	0.50	0.50	0.50	0.50
L‐lysine	0.50	0.50	0.50	0.50	0.50	0.50
Salt	0.25	0.25	0.25	0.25	0.25	0.25
Ascorbic acid	0.01	0.01	0.01	0.01	0.01	0.01
Total	100	100	100	100	100	100
Analysed proximate composition						
Dry matter (DM; g/100 g diet)	89.80	89.75	90.00	90.06	89.86	90.15
Crude protein (CP; g/100 g DM)	35.14	35.04	35.05	34.75	35.09	35.09
Crude fat (EE; g/100 g DM)	11.00	11.01	10.95	11.03	10.96	11.00
Crude fibre (CF; g/100 g DM)	5.01	4.99	4.99	5.00	4.99	4.98
Ash (g/100 g DM)	7.02	7.05	7.00	7.01	6.98	6.96
Carbohydrate (g/100 g DM)	41.83	41.92	42.01	42.21	41.97	41.96
Gross energy (kcal/100 g DM)	492.70	492.43	492.34	492.25	492.57	492.86

*Note:* D0.36, D0.96, D1.05, D1.15, D1.23 and D1.41 represent the six experimental diets formulated by including 0%, 0.67%, 0.77%, 0.87%, 0.97% and 1.17% methionine, respectively, in place of cellulose, resulting in analysed dietary methionine concentrations of 0.36%, 0.96%, 1.05%, 1.15%, 1.23% and 1.41%, respectively. Composition per kg of premix: vitamin A—3,000,000 IU, vitamin D3—700,000 IU, vitamin E (alpha‐tocopherol)—3500 mg, vitamin C (heat‐stable L‐ascorbyl‐2‐polyphosphate)—20,000 mg, vitamin K3 (menadione)—2400 mg, vitamin B1 (thiamine)—1500 mg, vitamin B2 (riboflavin)—3500 mg, vitamin B6 (pyridoxine)—400 mg, vitamin B12 (cyanocobalamin)—10 mg, vitamin B3 (niacin)—10,000 mg, pantothenic acid (Ca‐D‐pantothenate)—5000 mg, folic acid—500 mg, biotin—9.5 mg, choline chloride—40,000 mg, iron (Fe)—18,750 mg, zinc (Zn)—12,500 mg, manganese (Mn)—6000 mg, copper (Cu)—1500 mg, iodine (I)—250 mg, cobalt (Co)—250 mg and selenium (Se)—50 mg.

Preparation of the feeds involved milling the dry ingredients of each experimental diet using a hammer mill (GM35—Ndume Limited Company, Kenya) and weighing (WSS S.S Waterproof Baker Dough Scale, Model WSS6) to meet the required compositions of the respective diets. Using MS Excel, six diets were formulated with pre‐standardised nutrient composition of each ingredient. The mixture was thoroughly mixed using a mixer (Waring LBC15 Stainless Steel Laboratory Blender, 1 gal, 120 V/60 Hz); then, soybean oil and warm water (15%–20% of the diet mixture) of about 40°C were added to make dough, which was extruded with a DSE32 Lab Extruder (diameter = 1.9 mm, length = 2 mm; die number 9) to make pellets. The pellets were dried for 6–8 h in the shade to avoid direct sunlight, which could degrade their nutritional value, particularly fats and vitamins. A total of 10 kg of each diet was prepared, sealed in labelled zipper bags, and stored at −20°C until use.

At the start of the study, 50 g of each formulated diet were taken for nutritional analysis to determine their proximate (Table 1) and amino acid (Table 2) composition at the *icipe* nutritional analysis laboratory in Nairobi, Kenya. Dry matter (DM; g/100 g diet), CP (g/100 g DM), crude fat/ether extract (EE; g/100 g DM), crude fibre (CF; g/100 g DM), ash (g/100 g DM) and carbohydrate (g/100 g DM) content were determined using standard protocols [29]. DM content was determined by oven‐drying the sample at 105°C for 20 h and recording the resulting moisture loss. CP was analysed using the Kjeldahl method. Crude fat, expressed as EE, was determined using the Soxhlet extraction technique with ether as the solvent. CF was measured by sequentially boiling the sample in 1.3% sulphuric acid (H_2_SO_4_), followed by 1.3% sodium hydroxide (NaOH). Ash content was determined by incinerating the sample in a muffle furnace at 550°C until a constant weight was obtained, and the resulting residue was expressed as a percentage of the dry sample weight. The carbohydrate content, expressed as the nitrogen‐free extract (NFE), was calculated by deducting the percentages of all other proximate components from the total composition of 100%, as shown below:

**Table 2:** Amino acid profile of the experimental diets fed for 84 days to the local and improved Nile tilapia strains.

Amino acids (g/100 g DM)	Experimental diets					
	D0.36	D0.96	D1.05	D1.15	D1.23	D1.41
Essential amino acids						
Arginine	2.44	2.45	2.42	2.46	2.44	2.41
Histidine	0.88	0.88	0.87	0.87	0.87	0.87
Isoleucine	1.55	1.56	1.58	1.60	1.57	1.56
Leucine	3.14	3.13	3.10	3.15	3.15	3.18
Lysine	2.10	2.09	2.11	2.10	2.09	2.08
Methionine	0.36	0.96	1.05	1.15	1.23	1.41
Phenylalanine	1.58	1.57	1.58	1.60	1.62	1.58
Threonine	1.39	1.40	1.37	1.38	1.39	1.41
Tryptophan	0.38	0.41	0.37	0.40	0.42	0.36
Valine	1.76	1.74	1.74	1.75	1.76	1.74
Non‐essential amino acids						
Alanine	1.53	1.54	1.54	1.56	1.58	1.56
Aspartic acid	3.52	3.49	3.49	3.50	3.51	3.50
Cysteine	0.42	0.42	0.42	0.42	0.42	0.42
Glutamic acid	5.25	5.22	5.25	5.23	5.23	5.24
Glycine	1.58	1.57	1.58	1.56	1.57	1.58
Proline	1.75	1.76	1.74	1.75	1.75	1.74
Serine	1.40	1.39	1.41	1.38	1.41	1.40
Tyrosine	1.21	1.08	1.22	1.09	1.24	1.08

*Note:* D0.36, D0.96, D1.05, D1.15, D1.23 and D1.41 represent the six experimental diets formulated by including 0%, 0.67%, 0.77%, 0.87%, 0.97% and 1.17% methionine, respectively, in place of cellulose, resulting in analysed dietary methionine concentrations of 0.36%, 0.96%, 1.05%, 1.15%, 1.23% and 1.41%, respectively.

Total carbohydrates=100−CP+EE+CF+ash.

Gross energy (GE; kcal/100 g DM) was calculated according to the NRC [28] as follows:

GE=CP×5.6+EE×9.44+4.1×CF+ NFE.

The amino acid composition of the feed samples was determined following the procedure described by Otter [30]. In brief, homogenised feed samples were subjected to performic acid oxidation prior to acid hydrolysis to stabilise the sulphur‐containing amino acids. The oxidised samples were subsequently hydrolysed in 10 mL of 6 N HCl at 110°C for 24 h. Following hydrolysis, the individual amino acids were separated, identified, and quantified using liquid chromatography–mass spectrometry (LC–MS).

### 2.4. Experimental Fish and Feeding

A batch of 1260 all‐male tilapia fingerlings was acquired for the study. The improved strain was obtained from the Kenya Marine and Fisheries Research Institute (KMFRI), Sagana Research Centre, Kenya, where *O. niloticus* has undergone eight generations of selective breeding to produce the Sagana strain [24]. The local/unimproved strain was acquired from Mwea Fish Farm in Kenya, where no genetic improvement has been carried out. The farm acquired its original stock from KMFRI Sagana before undergoing any form of genetic improvement. This was done to ensure that the improved strain and the local strain share the same genetic origin and that any observed differences in nutrient requirements are due to the experimental diets and genetic improvement rather than unrelated genetic background differences.

The fingerlings were transported to *icipe* in oxygenated polyethylene bags. On arrival at *icipe*, an equal number of fish were distributed to four (4) holding plastic tanks and acclimatised for 2 weeks, during which the fish were fed a commercial feed (0.8 mm Nutra diet). The day before the start of the experiment, fish were starved to empty their digestive systems and obtain the actual fish weight and to reduce the stress of handling at stocking. Each strain of fish was then pooled into 2 m^3^ tanks to mix and then randomly distributed to the experimental tanks. Thirty‐five (35) fish were stocked in each 100 L tank at approximately the same starting average weight (4.08 ± 0.12 g) to commence the experiment, which (assuming the tank contains 95 L of water) represents a stocking density of 368 fish per m^3,^ which is within the recommended stocking density for *O. niloticus* juveniles in RAS [31, 32].

Any fish that died in the first 2–3 days after stocking were replaced, seeing it as mortality due to the stress of stocking, but any mortality observed after that was recorded as mortality due to experimentation. During the experiment, fish mortality was recorded daily if any. Fish were fed manually to satiation twice daily [33], in the morning (09:00–10:00 h) and in the evening (16:00–17:00 h). At first feeding, the feed was crushed into smaller particles to enable the fish to feed actively since the fish could not take the 2 mm feed size prepared. The amount of feed given in each tank was recorded daily.

### 2.5. Measurement of Water Quality Parameters

Water quality parameters, including temperature, DO and pH, were monitored using a portable handheld multi‐parameter water quality metre (HI98194, Hanna Instruments, Smithfield, Rhode Island, USA). Measurements of temperature, pH and DO were taken daily at 11:30 h (~2 h after morning feeding) from 10 randomly selected tanks and the sump. During the experimental period, water temperature was maintained at 26–28°C, DO at 5–7 mg/L and pH at 6.5–7.5. For the analysis of nitrite (NO_2_^−^), nitrate (NO_3_^−^) and ammonia (NH_3_), 100 mL of water samples were collected weekly and analysed in the laboratory using a UV‐1800 Shimadzu spectrophotometer (Shimadzu Corporation, Tokyo, Japan) following standard colorimetric procedures. The NO_2_^−^, NO_3_^−^ and NH_3_ levels were maintained at 0.01–0.3, 1–2 and 0.01–0.04 mg/L, respectively, throughout the experiment.

### 2.6. Sampling of Fish

After every 2 weeks, 30 fish from each tank were individually weighed and measured for length without anaesthesia to monitor growth performance and to calculate other growth parameters. The day before the sampling, fish were not fed to allow them to empty their guts to obtain the accurate fish weight and to reduce handling stress during sampling. At the end of the growth trials (84 days), three fish from each experimental tank were randomly selected and anaesthetised using clove oil at a rate of 90 mg/L [34] for blood collection (serum biochemical, hematological and antioxidant analyses), dissected, their liver and viscera were weighed to calculate the hepatosomatic and viscerosomatic indices and their carcase was dried for amino acid analyses.

### 2.7. Determination of the Growth Performance, Feed Utilisation and Somatic Indices

After each feeding session, any uneaten feed was recovered from each tank through siphoning, placed in labelled plastic zipper bags and stored at −20°C. At the end of the biweekly fish sampling, the collected feed residues were dried and weighed to enable precise determination of the apparent feed intake. The parameters that were calculated from the growth and feed intake data to determine the growth performance, feed utilisation and somatic indices include weight gain (g), feed conversion ratio (FCR), specific growth rate (SGR; % g day^−1^), feed efficiency ratio (FER), protein efficiency ratio (PER), hepatosomatic index (HSI) and viscerosomatic index (VSI) in each tank. Fish survival (%) was determined by dividing the final number of fish by the initial number of fish and multiplying the result by 100 for each experimental tank.

The parameters were calculated as follows:

Weight gaing= Final body weightg−initial body weightg.

Daily weight gain= Weight gain/days.

Feed conversion ratioFCR=Feed intake g/weight gain g.

Specific growth rate SGR,%=100×ln final weightg− ln initial weightg/days.

Condition factor g/cm3=100×weight g/length cm3.

Survival rate %=100×final number of fish/initial number of fish.

Feed efficiency ratio FER= Weight gain g/total feed intake g.

Protein efficiency ratio PER= Weight gain g/protein feed intake g.

Viscerosomatic index VSI;%=100×visceral weight g/body weight g.

Hepatosomatic index HSI;%=100×liver weight g/body weight g.

### 2.8. Determination of Fish Haemato‐Biochemical and Antioxidant Biomarkers

At the end of the experiment, the fish were fasted for 24 h before blood sampling, and three fish from each tank were anaesthetised using clove oil. Blood samples were obtained from the caudal vein using heparinised needles (0.6–1.2 mm diameter, depending on fish size) and transferred into two sets of vacutainer tubes. One set contained ethylenediaminetetraacetic acid (EDTA; 1.8 mg/mL of whole blood) as an anticoagulant for haematology, and another set with no anticoagulant to clot at 4°C.

#### 2.8.1. Serum Biochemical Analyses

The samples were centrifuged at 3000 rpm for 15 min at room temperature to separate the serum, which was used for biochemical and antioxidant analyses. The serum biochemical parameters, including total protein, albumin (g/dL), triglycerides (mg/dL), high‐density lipoprotein (HDL; mg/dL), low‐density lipoprotein (LDL; mg/dL), free fatty acids (FFAs; nmol/L), glucose (mg/dL), cholesterol (mg/dL), cortisol (ng/mL), blood urea nitrogen (BUN; mg/dL), aspartate aminotransferase (AST; U/L) and alanine aminotransferase (ALT; U/L) levels were determined using automatic biochemical analyser (Sysmex BX‐3010, Sysmex Corporation, Kobe, Japan). Globulin (g/dL) levels were calculated by subtracting the measured albumin levels from the total protein levels:

Globulin= Total protein−albumin.

#### 2.8.2. Serum Antioxidant Assays

The activities of superoxide dismutase (SOD, U/mL protein) and glutathione peroxidase (GSH‐Px, U/mL protein), as well as malondialdehyde (MDA, nmol/mL protein) levels in serum, were determined using colorimetric methods with commercial kits (Nanjing Jiancheng Bioengineering Institute, Nanjing, China) following Zhou et al. [35]. SOD activity was measured at 550 nm on a UV–visible spectrophotometer based on the reduction of nitrite by a xanthine–xanthine oxidase system, which generates superoxide anions. GSH‐Px activity was assessed by monitoring the decrease in glutathione, as indicated by a change in absorbance at 412 nm. MDA levels were determined by reacting the serum supernatant with thiobarbituric acid (TBA) to produce a pink‐coloured complex, which was heated in a water bath at 95°C for 45 min, cooled to room temperature, and then measured at 532 nm using a UV–visible spectrophotometer.

#### 2.8.3. Haematological Analyses

Red blood cells (RBCs; 10^6^/µL), haematocrit (Hct; %), haemoglobin (Hgb; g/dL), white blood cells (WBCs; 10^3^/µL), platelet count (10^3^/µL), lymphocytes (10^3^/µL), monocytes (10^3^/µL) and neutrophils (%) were measured using an automated haematology analyser (Sysmex XN‐1000, Sysmex Corporation, Kobe, Japan).

The mean corpuscular volume (MCV; fL), mean corpuscular Hgb (MCH; pg), and mean corpuscular Hgb concentration (MCHC; g/dL) were calculated as:

Mean corpuscular volume MCV=Haematocrit/RBC count×10.

Mean corpuscular haemoglobin (MCH)=Haemoglobin/RBC count×10.

Mean corpuscular haemoglobin concentration (MCHC)=Haemoglobin/haematocrit×100.

### 2.9. Carcase Amino Acid Analysis

After the experimental period, three fish per tank were randomly selected, eviscerated, freeze‐dried and ground to evaluate the carcase amino acid composition following the procedure by Otter [30] as described in Section 2.3.

### 2.10. Statistical Analyses

The data were subjected to a two‐way ANOVA in R Version 4.2.1 [36] to determine the effects of dietary methionine inclusion levels between the two *O. niloticus* strains. To determine differences in growth performance, somatic indices, feed utilisation and the serum biochemical and antioxidant capacity between the strains, the data were analysed using a two‐way ANOVA with strain (local vs. improved) and methionine level as fixed factors. All parameters were treated as dependent variables and analysed separately. Prior to analysis, data were checked for normality (Shapiro–Wilk test) and homoscedasticity (Levene’s test) using the *car* package in R [37]. The ANOVA was fitted, and where significant effects were detected, pairwise comparisons were conducted using Tukey’s honestly significant difference (HSD) using the *emmeans* package in R [38]. The results were presented as means ± standard error and visualised with plots using the *ggplot2* package in R [39].

The data were subjected to quadratic regression analysis to estimate the optimal dietary methionine requirement for the two strains of *O. niloticus*. For each selected variable, that is, weight gain, SGR and FCR, the methionine level was treated as a continuous predictor, while the response variable was fitted to a quadratic model. The quadratic peak (vertex), representing the optimal methionine level, was estimated from the model parameters. Model diagnostics (normality, residual plots and leverage) were examined using the *performance* package in R [40]. The visualisation of model fits and estimated optima was generated using the *ggplot2* package in R, including scatterplots with fitted quadratic curves [39].

## 3. Results

### 3.1. Growth Performance, Feed Utilisation and Somatic Indices

The local strain had the highest weight gain at an inclusion of methionine at 10.5 g/kg of the diet (D1.05) over 84 days of the growth trial, while the improved strain had the highest weight gain at an inclusion of methionine at 11.5 g/kg of the diet (D1.15), as shown in Figure 1.

**Figure 1:**
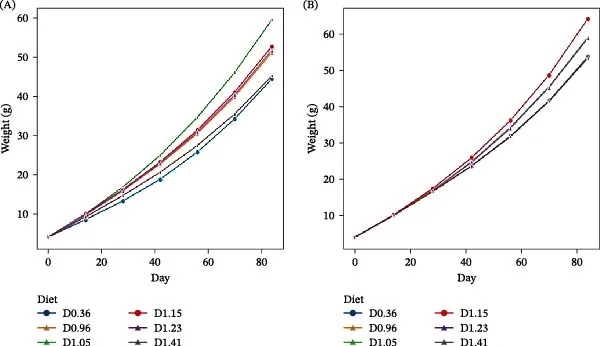
Growth curves of local (A) and improved (B) strains of Nile tilapia fed experimental diets containing graded levels of methionine for 84 days.

The initial weights did not vary significantly among the treatments for both strains (*p* > 0.05), implying homogeneity at the start of the trial (Table 3). The growth performance and feed utilisation parameters were significantly influenced by dietary methionine, strain and their interaction (*p* < 0.05). For the local strain, an inclusion of methionine at 10.5 g/kg of the diet (D1.05) resulted in significantly higher (*p* < 0.05) weight gain (55.4 ± 4.10 g), daily weight gain (0.660 ± 0.01 g), SGR (3.19 ± 0.01), FER (0.595 ± 0.01) and PER (1.58 ± 0.01), and significantly lower (*p* < 0.05) FCR (1.68 ± 0.01) than the other treatments (*p* < 0.05), with a significant difference among the treatments (*p* < 0.05). For the improved strain, an inclusion of methionine at 11.5 g/kg of the diet (D1.15) resulted in significantly higher (*p* < 0.05) weight gain (60.4 ± 7.24 g), daily weight gain (0.719 ± 0.02 g), SGR (3.28 ± 0.01), FER (0.625 ± 0.01) and PER (1.74 ± 0.01), and significantly lower (*p* < 0.05) FCR (1.60 ± 0.01), with a significant variation among the treatments (*p* < 0.05).

**Table 3:** Growth performance and feed utilisation of the local and improved strains of Nile tilapia fed experimental diets containing graded levels of methionine for 84 days.

Diet	Strain	Parameters								
		Initial weight (g)	Final weight (g)	Weight gain (g)	Daily weight gain (g)	Feed intake (g)	FCR	SGR (%)	FER	PER
D0.36	Local	4.09 ± 0.11^a^	44.4 ± 6.12^e^	40.3 ± 5.02^d^	0.480 ± 0.03^c^	78.5 ± 5.07^d^	1.95 ± 0.01^a^	2.84 ± 0.01^c^	0.513 ± 0.01^e^	1.15 ± 0.01^c^
D0.96	Local	4.04 ± 0.05^a^	51.1 ± 5.08^c^	47.1 ± 4.90^c^	0.561 ± 0.01^b^	85.9 ± 6.28^b^	1.82 ± 0.01^b^	3.02 ± 0.01^b^	0.548 ± 0.01^d^	1.34 ± 0.02^b^
D1.05	Local	4.08 ± 0.04^a^	59.5 ± 6.11^a^	55.4 ± 4.10^a^	0.660 ± 0.01^a^	93.1 ± 5.03^a^	1.68 ± 0.01^d^	3.19 ± 0.01^a^	0.595 ± 0.01^a^	1.58 ± 0.01^a^
D1.15	Local	4.07 ± 0.19^a^	52.7 ± 6.08^b^	48.6 ± 6.11^b^	0.579 ± 0.02^b^	83.4 ± 6.35^c^	1.72 ± 0.01^c^	3.05 ± 0.01^b^	0.583 ± 0.01^b^	1.40 ± 0.02^b^
D1.23	Local	4.10 ± 0.14^a^	51.7 ± 5.11^c^	47.6 ± 7.58^c^	0.567 ± 0.03^b^	84.7 ± 5.29^b^	1.78 ± 0.01^c^	3.02 ± 0.01^b^	0.562 ± 0.01^c^	1.36 ± 0.01^b^
D1.41	Local	4.08 ± 0.08^a^	45.1 ± 5.11^d^	41.0 ± 5.90^d^	0.489 ± 0.03^c^	77.2 ± 5.13^d^	1.88 ± 0.01^b^	2.86 ± 0.01^c^	0.532 ± 0.01^d^	1.17 ± 0.01^c^
D0.36	Improved	4.09 ± 0.11^a^	54.0 ± 5.02^c^	49.9 ± 5.18^c^	0.594 ± 0.03^c^	93.5 ± 6.42^b^	1.87 ± 0.01^a^	3.07 ± 0.01^c^	0.534 ± 0.01^e^	1.42 ± 0.01^c^
D0.96	Improved	4.09 ± 0.05^a^	59.0 ± 5.15^b^	54.9 ± 7.01^b^	0.654 ± 0.01^b^	93.7 ± 6.30^b^	1.71 ± 0.01^b^	3.18 ± 0.02^b^	0.586 ± 0.01^c^	1.57 ± 0.01^b^
D1.05	Improved	4.08 ± 0.06^a^	59.3 ± 5.06^b^	55.3 ± 6.11^b^	0.658 ± 0.04^b^	90.4 ± 5.24^c^	1.64 ± 0.01^c^	3.19 ± 0.02^b^	0.611 ± 0.01^b^	1.58 ± 0.01^b^
D1.15	Improved	4.09 ± 0.19^a^	64.5 ± 6.33^a^	60.4 ± 7.24^a^	0.719 ± 0.02^a^	96.6 ± 6.11^a^	1.60 ± 0.01^d^	3.28 ± 0.01^a^	0.625 ± 0.01^a^	1.74 ± 0.01^a^
D1.23	Improved	4.07 ± 0.05^a^	59.1 ± 5.05^b^	55.0 ± 7.30^b^	0.655 ± 0.01^b^	90.6 ± 5.12^c^	1.65 ± 0.01^c^	3.19 ± 0.01^b^	0.607 ± 0.01^b^	1.57 ± 0.01^b^
D1.41	Improved	4.10 ± 0.12^a^	53.6 ± 5.12^c^	49.5 ± 5.08^c^	0.589 ± 0.03^c^	90.1 ± 6.51^c^	1.82 ± 0.01^a^	3.06 ± 0.01^c^	0.549 ± 0.01^d^	1.41 ± 0.01^c^
Diet										
D0.36	—	4.09 ± 0.08^a^	49.19 ± 4.37^c^	45.09 ± 5.73^d^	0.54 ± 0.03^c^	85.99 ± 6.47^d^	1.91 ± 0.01^a^	2.95 ± 0.03^c^	0.52 ± 0.01^d^	1.29 ± 0.01^c^
D0.96	—	4.07 ± 0.19^a^	55.08 ± 6.82^b^	51.01 ± 6.92^c^	0.61 ± 0.01^b^	89.78 ± 5.46^b^	1.76 ± 0.01^c^	3.10 ± 0.03^b^	0.57 ± 0.01^bc^	1.46 ± 0.01^b^
D1.05	—	4.08 ± 0.11^a^	59.41 ± 5.01^a^	55.33 ± 5.02^a^	0.66 ± 0.02^a^	91.73 ± 5.28^a^	1.66 ± 0.01^e^	3.19 ± 0.02^a^	0.60 ± 0.01^a^	1.59 ± 0.02^a^
D1.15	—	4.08 ± 0.08^a^	58.58 ± 6.82^a^	54.50 ± 5.37^b^	0.65 ± 0.02^a^	90.01 ± 6.02^ab^	1.66 ± 0.01^e^	3.16 ± 0.02^a^	0.60 ± 0.01^a^	1.56 ± 0.01^a^
D1.23	—	4.09 ± 0.12^a^	55.39 ± 6.38^b^	51.31 ± 6.36^c^	0.61 ± 0.03^b^	87.66 ± 6.04^c^	1.71 ± 0.01^d^	3.10 ± 0.01^b^	0.58 ± 0.01^ab^	1.47 ± 0.01^b^
D1.41	—	4.09 ± 0.08^a^	49.34 ± 5.30^c^	45.26 ± 5.28^d^	0.54 ± 0.01^c^	83.66 ± 5.46^e^	1.85 ± 0.01^b^	2.96 ± 0.03^c^	0.54 ± 0.01^c^	1.30 ± 0.02^c^
Strain										
Local	—	4.08 ± 0.11^a^	50.75 ± 5.50^b^	46.68 ± 6.38^b^	0.56 ± 0.03^b^	83.79 ± 5.34^b^	1.81 ± 0.01^a^	3.00 ± 0.08^b^	0.55 ± 0.01^b^	1.34 ± 0.02^b^
Improved	—	4.09 ± 0.05^a^	58.24 ± 6.38^a^	54.15 ± 5.37^a^	0.64 ± 0.02^a^	92.48 ± 6.02^a^	1.71 ± 0.01^b^	3.16 ± 0.11^a^	0.58 ± 0.01^a^	1.55 ± 0.02^a^
Two‐way ANOVA										
Diet	—	NS	^∗∗∗^	^∗∗∗^	^∗∗∗^	^∗∗∗^	^∗∗∗^	^∗∗∗^	^∗∗∗^	^∗∗∗^
Strain	—	NS	^∗∗∗^	^∗∗∗^	^∗∗∗^	^∗∗∗^	^∗∗∗^	^∗∗∗^	^∗∗∗^	^∗∗∗^
Diet × strain	—	NS	^∗∗∗^	^∗∗∗^	^∗∗∗^	^∗∗∗^	^∗∗∗^	^∗∗∗^	^∗∗∗^	^∗∗∗^

*Note:* D0.36, D0.96, D1.05, D1.15, D1.23 and D1.41 represent the six experimental diets formulated by including 0%, 0.67%, 0.77%, 0.87%, 0.97% and 1.17% methionine, respectively, in place of cellulose, resulting in analysed dietary methionine concentrations of 0.36%, 0.96%, 1.05%, 1.15%, 1.23% and 1.41%, respectively. Values given as mean ± SE. Values with different lowercase superscript letters within columns are significantly different (*p* < 0.05). Two‐way ANOVA: NS = non‐significant (*p* > 0.05),  ^∗∗∗^ *p* < 0.001.

Abbreviations: FCR, feed conversion ratio; FER, feed efficiency ratio; PER, protein efficiency ratio; SGR, specific growth rate.

There was a significant effect of dietary methionine, strain and their interaction on the condition factor, HSI and VSI (*p* < 0.05). However, there was no significant effect of the strain and the interaction between the strain and dietary methionine on the survival rate (*p* > 0.05). The significantly higher (*p* < 0.05) condition factor (1.98 ± 0.01), survival rate (96.19 ± 6.00%) and HSI (1.24 ± 0.01%), and significantly lower (*p* < 0.05) VSI (5.46% ± 0.08%) for the local strain were recorded at an inclusion of methionine at 10.5 g/kg of the diet (D1.05), after which the levels declined (Table 4). For the improved strain, the significantly higher (*p* < 0.05) condition factor (1.95 ± 0.01), survival rate (96.19% ± 6.35%), and HSI (1.38% ± 0.01%), and significantly lower (*p* < 0.05) VSI (5.29% ± 0.10%) were recorded at an inclusion of methionine at 11.5 g/kg of the diet (D1.15), after which the levels declined.

**Table 4:** The condition factor, survival rate, hepatosomatic index (HSI) and viscerosomatic index (VSI) of the local and improved strains of Nile tilapia fed experimental diets containing graded levels of methionine for 84 days.

Diet	Strain	Parameters			
		Condition factor	Survival rate (%)	HSI (%)	VSI (%)
D0.36	Local	1.25 ± 0.01^d^	88.57 ± 6.18^d^	1.14 ± 0.01^bc^	5.61 ± 0.11^a^
D0.96	Local	1.70 ± 0.01^b^	93.33 ± 6.28^b^	1.22 ± 0.02^ab^	5.53 ± 0.10^c^
D1.05	Local	1.98 ± 0.01^a^	96.19 ± 6.00^a^	1.24 ± 0.01^a^	5.46 ± 0.08^d^
D1.15	Local	1.61 ± 0.10^c^	92.38 ± 5.12^bc^	1.19 ± 0.01^b^	5.50 ± 0.11^c^
D1.23	Local	1.15 ± 0.01^e^	90.48 ± 6.08^cd^	1.12 ± 0.01^c^	5.52 ± 0.11^c^
D1.41	Local	1.12 ± 0.01^e^	87.62 ± 5.41^d^	1.00 ± 0.01^d^	5.58 ± 0.11^b^
D0.36	Improved	1.39 ± 0.01^e^	87.62 ± 5.48^d^	1.18 ± 0.02^e^	5.40 ± 0.08^a^
D0.96	Improved	1.64 ± 0.01^c^	91.43 ± 6.01^c^	1.32 ± 0.01^c^	5.34 ± 0.10^b^
D1.05	Improved	1.58 ± 0.01^d^	93.33 ± 5.05^b^	1.34 ± 0.02^bc^	5.30 ± 0.10^c^
D1.15	Improved	1.95 ± 0.01^a^	96.19 ± 6.35^a^	1.38 ± 0.01^a^	5.29 ± 0.10^d^
D1.23	Improved	1.75 ± 0.01^b^	92.38 ± 5.21^bc^	1.28 ± 0.03^d^	5.32 ± 0.11^b^
D1.41	Improved	1.53 ± 0.01^d^	89.52 ± 5.01^cd^	1.13 ± 0.02^f^	5.43 ± 0.11^a^
Diet					
D0.36	—	1.32 ± 0.01^d^	88.09 ± 5.07^b^	1.16 ± 0.01^c^	5.51 ± 0.11^a^
D0.96	—	1.67 ± 0.01^b^	92.38 ± 5.63^ab^	1.27 ± 0.01^a^	5.43 ± 0.10^b^
D1.05	—	1.78 ± 0.01^a^	94.76 ± 6.20^a^	1.29 ± 0.02^a^	5.38 ± 0.11^c^
D1.15	—	1.78 ± 0.01^a^	94.28 ± 5.21^a^	1.28 ± 0.03^a^	5.39 ± 0.08^c^
D1.23	—	1.45 ± 0.01^c^	91.43 ± 6.64^b^	1.20 ± 0.02^b^	5.42 ± 0.11^b^
D1.41	—	1.32 ± 0.01^d^	88.57 ± 6.25^b^	1.06 ± 0.02^d^	5.51 ± 0.11^a^
Strain					
Local	—	1.47 ± 0.01^b^	91.43 ± 6.12^a^	1.15 ± 0.02^b^	5.53 ± 0.08^a^
Improved	—	1.64 ± 0.01^a^	91.75 ± 6.20^a^	1.27 ± 0.02^a^	5.35 ± 0.11^b^
Two‐way ANOVA					
Diet	—	^∗∗∗^	^∗∗∗^	^∗∗∗^	^∗∗^
Strain	—	^∗∗∗^	NS	^∗∗∗^	^∗∗∗^
Diet × strain	—	^∗∗∗^	NS	^∗∗^	^∗∗∗^

*Note:* D0.36, D0.96, D1.05, D1.15, D1.23 and D1.41 represent the six experimental diets formulated by including 0%, 0.67%, 0.77%, 0.87%, 0.97% and 1.17% methionine, respectively, in place of cellulose, resulting in analysed dietary methionine concentrations of 0.36%, 0.96%, 1.05%, 1.15%, 1.23% and 1.41%, respectively. Values given as mean ± SE. Values with different lowercase superscript letters within columns are significantly different (*p* < 0.05). Two‐way ANOVA: NS = non‐significant (*p* > 0.05),  ^∗∗^ *p* < 0.005,  ^∗∗∗^ *p* < 0.001.

### 3.2. Serum Biochemical Indices

There was a significant effect of dietary methionine, strain and their interaction on all serum biochemical indices (*p* < 0.05), except for the strain effect on albumin levels (*p* > 0.05). As presented in Table 5, significantly higher (*p* < 0.05) levels of protein (3.00 ± 0.02 g/dL), albumin (1.5 ± 0.01 g/dL), globulin (1.49 ± 0.03 g/dL) and HDL (46.7 ± 4.06 mg/dL), and significantly lower (*p* < 0.05) levels of triglycerides (91.4 ± 13.58 mg/dL), LDL (101.2 ± 12.80 mg/dL), FFA (0.45 ± 0.01 nmol/L) and glucose (85.3 ± 8.80 mg/dL), were recorded at an inclusion of methionine at 10.5 g/kg of the diet (D1.05) for the local strain. For the improved strain, significantly higher (*p* < 0.05) levels of protein (4.51 ± 0.03 g/dL), albumin (1.5 ± 0.01 g/dL), globulin (2.98 ± 0.02 g/dL) and HDL (56.7 ± 4.58 mg/dL), and significantly lower (*p* < 0.05) levels of triglycerides (117.5 ± 10.00 mg/dL), LDL (72.7 ± 8.40 mg/dL), FFA (0.33 ± 0.02 nmol/L) and glucose (56.7 ± 7.45 mg/dL) were recorded at an inclusion of methionine at 11.5 g/kg of the diet (D1.15).

**Table 5:** Serum total protein, albumin, globulin, triglycerides, high‐density lipoprotein (HDL), low‐density lipoprotein (LDL), free fatty acids (FFA) and glucose levels of the local and improved strains of Nile tilapia fed experimental diets containing graded levels of methionine for 84 days.

Diet	Strain	Parameters							
		Protein (g/dL)	Albumin (g/dL)	Globulin (g/dL)	Triglycerides (mg/dL)	HDL (mg/dL)	LDL (mg/dL)	FFA (nmol/L)	Glucose (mg/dL)
D0.36	Local	2.58 ± 0.04^c^	1.2 ± 0.02^c^	1.38 ± 0.01^d^	116.9 ± 13.88^a^	37.0 ± 3.57^e^	126.1 ± 13.88^a^	0.61 ± 0.01^a^	93.3 ± 7.29^b^
D0.96	Local	2.73 ± 0.03^b^	1.3 ± 0.01^b^	1.43 ± 0.03^b^	108.6 ± 14.58^c^	39.7 ± 3.34^c^	117.3 ± 13.57^c^	0.57 ± 0.02^b^	89.3 ± 7.85^d^
D1.05	Local	3.00 ± 0.02^a^	1.5 ± 0.01^a^	1.49 ± 0.03^a^	91.4 ± 13.58^f^	46.7 ± 4.06^a^	101.2 ± 12.80^f^	0.45 ± 0.01^e^	85.3 ± 8.80^f^
D1.15	Local	2.73 ± 0.03^b^	1.3 ± 0.01^b^	1.43 ± 0.02^b^	104.3 ± 10.58^d^	40.7 ± 3.33^b^	114.0 ± 10.55^d^	0.53 ± 0.01^c^	87.3 ± 8.52^e^
D1.23	Local	2.72 ± 0.02^b^	1.3 ± 0.04^b^	1.42 ± 0.01^b^	99.3 ± 11.33^e^	38.7 ± 3.58^d^	101.3 ± 11.88^e^	0.49 ± 0.01^d^	90.7 ± 8.80^c^
D1.41	Local	2.59 ± 0.03^c^	1.2 ± 0.03^c^	1.39 ± 0.02^d^	113.0 ± 12.88^b^	30.7 ± 3.57^f^	123.0 ± 10.50^b^	0.61 ± 0.01^a^	95.0 ± 7.39^a^
D0.36	Improved	3.89 ± 0.04^d^	1.3 ± 0.01^c^	2.60 ± 0.03^d^	135.6 ± 13.34^a^	47.7 ± 4.33^e^	83.7 ± 8.33^b^	0.42 ± 0.02^a^	69.7 ± 7.83^a^
D0.96	Improved	3.99 ± 0.02^c^	1.3 ± 0.01^c^	2.68 ± 0.02^c^	129.8 ± 13.38^b^	51.3 ± 4.33^d^	79.3 ± 9.58^c^	0.37 ± 0.01^b^	64.3 ± 8.57^b^
D1.05	Improved	4.20 ± 0.04^b^	1.4 ± 0.02^b^	2.83 ± 0.01^b^	125.3 ± 12.38^c^	53 ± 4.58^b^	77.0 ± 8.57^d^	0.36 ± 0.01^b^	60.3 ± 8.85^c^
D1.15	Improved	4.51 ± 0.03^a^	1.5 ± 0.01^a^	2.98 ± 0.02^a^	117.5 ± 10.00^e^	56.7 ± 4.58^a^	72.7 ± 8.40^e^	0.33 ± 0.02^c^	56.7 ± 7.45^d^
D1.23	Improved	4.14 ± 0.03^b^	1.3 ± 0.02^c^	2.83 ± 0.02^b^	123.0 ± 10.30^d^	52.7 ± 4.33^c^	76.7 ± 7.58^d^	0.36 ± 0.02^b^	60.7 ± 7.30^c^
D1.41	Improved	3.73 ± 0.03^e^	1.2 ± 0.01^d^	2.52 ± 0.01^e^	135.7 ± 11.38^a^	47.7 ± 4.58^e^	84.7 ± 9.30^a^	0.42 ± 0.01^a^	69.7 ± 7.68^a^
Diet									
D0.36	—	3.23 ± 0.04^d^	1.3 ± 0.03^b^	1.96 ± 0.02^e^	126.3 ± 12.57^a^	42.3 ± 3.92^d^	104.9 ± 10.92^a^	0.51 ± 0.01^a^	81.5 ± 8.91^b^
D0.96	—	3.36 ± 0.02^c^	1.3 ± 0.02^b^	2.05 ± 0.03^d^	119.2 ± 13.88^c^	45.5 ± 3.28^c^	98.3 ± 11.38^c^	0.47 ± 0.01^b^	76.8 ± 7.82^c^
D1.05	—	3.60 ± 0.02^a^	1.4 ± 0.02^a^	2.16 ± 0.01^b^	108.3 ± 10.18^f^	49.8 ± 4.63^a^	89.1 ± 10.54^e^	0.41 ± 0.02^d^	72.8 ± 7.12^e^
D1.15	—	3.62 ± 0.03^a^	1.4 ± 0.01^a^	2.21 ± 0.02^a^	110.9 ± 11.30^e^	48.7 ± 3.09^b^	93.3 ± 11.73^d^	0.43 ± 0.01^c^	72.0 ± 8.74^e^
D1.23	—	3.43 ± 0.03^b^	1.3 ± 0.01^b^	2.09 ± 0.02^c^	111.2 ± 13.58^d^	45.7 ± 4.34^c^	89.0 ± 13.29^e^	0.42 ± 0.01^cd^	75.7 ± 7.29^d^
D1.41	—	3.16 ± 0.02^e^	1.2 ± 0.03^c^	1.99 ± 0.03^e^	124.4 ± 11.34^b^	39.2 ± 4.82^e^	103.8 ± 12.24^b^	0.51 ± 0.02^a^	82.3 ± 8.10^a^
Strain									
Local	—	2.73 ± 0.03^b^	1.3 ± 0.02^a^	1.41 ± 0.03^b^	105.6 ± 13.38^b^	38.9 ± 3.45^b^	113.8 ± 13.67^a^	0.54 ± 0.01^a^	90.1 ± 8.82^a^
Improved	—	4.08 ± 0.03^a^	1.3 ± 0.03^a^	2.75 ± 0.03^a^	127.8 ± 11.34^a^	51.5 ± 4.01^a^	79.0 ± 8.38^b^	0.38 ± 0.02^b^	63.6 ± 7.11^b^
Two‐way ANOVA									
Diet	—	^∗∗∗^	^∗∗∗^	^∗∗∗^	^∗∗∗^	^∗∗∗^	^∗∗∗^	^∗∗∗^	^∗∗∗^
Strain	—	^∗∗∗^	NS	^∗∗∗^	^∗∗∗^	^∗∗∗^	^∗∗∗^	^∗∗∗^	^∗∗∗^
Diet × strain	—	^∗∗∗^	^∗∗∗^	^∗∗∗^	^∗∗∗^	^∗∗∗^	^∗∗∗^	^∗∗∗^	^∗∗∗^

*Note:* D0.36, D0.96, D1.05, D1.15, D1.23 and D1.41 represent the six experimental diets formulated by including 0%, 0.67%, 0.77%, 0.87%, 0.97% and 1.17% methionine, respectively, in place of cellulose, resulting in analysed dietary methionine concentrations of 0.36%, 0.96%, 1.05%, 1.15%, 1.23% and 1.41%, respectively. Values given as mean ± SE. Values with different lowercase superscript letters within columns are significantly different (*p* < 0.05). Two‐way ANOVA:  ^∗^ *p* < 0.05,  ^∗∗∗^ *p* < 0.001.

Additionally, an inclusion of methionine at 10.5 g/kg of the diet (D1.05) showed significantly lower (*p* < 0.05) levels of serum cholesterol (159.0 ± 14.05 mg/dL), cortisol (27.7 ± 2.19 ng/mL), BUN (9.9 ± 1.06 mg/dL), AST (47.00 ± 4.11 U/L) and ALT (19.67 ± 2.05 U/L) for the local strain (Table 6). For the improved strain, significantly lower (*p* < 0.05) levels of serum cholesterol (130.9 ± 11.33 mg/dL), cortisol (27.2 ± 3.11 ng/mL), BUN (8.2 ± 1.00 mg/dL), AST (54.60 ± 4.05 U/L) and ALT (34.50 ± 3.10 U/L) were recorded at an inclusion of methionine at 11.5 g/kg of the diet (D1.15).

**Table 6:** Serum cholesterol, cortisol, blood urea nitrogen (BUN), aspartate aminotransferase (AST) and alanine aminotransferase (ALT) levels of the local and improved strains of Nile tilapia fed experimental diets containing different levels of methionine for 84 days.

Diet	Strain	Parameters				
		Cholesterol (mg/dL)	Cortisol (ng/mL)	BUN (mg/dL)	AST (U/L)	ALT (U/L)
D0.36	Local	185.8 ± 14.56^a^	41.0 ± 3.09^a^	12.3 ± 2.08^a^	61.00 ± 4.10^a^	27.67 ± 2.08^a^
D0.96	Local	176.6 ± 13.89^c^	35.7 ± 3.02^c^	11.7 ± 1.08^b^	57.67 ± 4.08^b^	25.67 ± 3.18^b^
D1.05	Local	159.0 ± 14.05^f^	27.7 ± 2.19^e^	9.9 ± 1.06^d^	47.00 ± 4.11^e^	19.67 ± 2.05^e^
D1.15	Local	171.3 ± 13.19^d^	32.7 ± 3.21^d^	11.1 ± 1.06^c^	54.67 ± 5.53^d^	23.67 ± 2.11^d^
D1.23	Local	166.5 ± 14.08^e^	33.7 ± 3.02^d^	11.0 ± 1.06^c^	55.67 ± 4.82^c^	24.67 ± 2.12^c^
D1.41	Local	184.1 ± 14.55^b^	39.7 ± 3.02^b^	12.2 ± 2.08^b^	61.33 ± 5.08^a^	27.67 ± 3.18^a^
D0.36	Improved	148.3 ± 13.58^a^	29.1 ± 3.18^a^	9.7 ± 1.10^a^	63.77 ± 5.75^a^	42.73 ± 3.08^a^
D0.96	Improved	141.2 ± 12.08^b^	28.3 ± 3.18^b^	9.0 ± 1.10^b^	59.80 ± 4.02^b^	38.67 ± 7.58^c^
D1.05	Improved	138.4 ± 12.58^c^	27.9 ± 3.01^c^	8.5 ± 1.05^c^	56.53 ± 4.58^d^	36.60 ± 3.00^d^
D1.15	Improved	130.9 ± 11.33^e^	27.2 ± 3.11^d^	8.2 ± 1.00^d^	54.60 ± 4.05^e^	34.50 ± 3.10^e^
D1.23	Improved	134.8 ± 13.46^d^	27.9 ± 2.19^c^	8.6 ± 1.10^c^	57.50 ± 5.45^c^	36.60 ± 3.56^d^
D1.41	Improved	148.6 ± 13.30^a^	28.9 ± 3.03^ab^	9.6 ± 1.05^ab^	63.50 ± 5.09^a^	42.60 ± 3.97^b^
Diet						
D0.36	—	167.1 ± 10.18^a^	35.1 ± 3.09^a^	11.0 ± 1.52^a^	62.38 ± 5.46^a^	35.20 ± 3.47^a^
D0.96	—	158.9 ± 13.24^c^	32.0 ± 3.53^c^	10.3 ± 1.04^b^	58.73 ± 4.09^b^	32.17 ± 3.92^b^
D1.05	—	148.7 ± 12.53^f^	27.8 ± 3.00^f^	9.2 ± 1.67^e^	51.76 ± 4.46^e^	28.13 ± 3.46^e^
D1.15	—	151.1 ± 12.40^d^	29.9 ± 2.45^e^	9.6 ± 1.36^d^	54.63 ± 5.36^d^	29.08 ± 3.46^d^
D1.23	—	150.6 ± 13.74^e^	30.8 ± 2.11^d^	9.8 ± 1.58^cd^	56.58 ± 4.52^c^	30.63 ± 3.70^c^
D1.41	—	166.4 ± 11.20^b^	34.3 ± 3.42^b^	10.9 ± 1.61^ab^	62.41 ± 5.64^a^	35.13 ± 3.86^a^
Strain						
Local	—	173.9 ± 13.46^a^	35.1 ± 2.95^a^	11.4 ± 1.74^a^	56.22 ± 4.86^b^	24.84 ± 3.07^b^
Improved	—	140.4 ± 13.46^b^	28.2 ± 3.35^b^	8.9 ± 1.05^b^	59.28 ± 4.66^a^	38.62 ± 3.55^a^
Two‐way ANOVA						
Diet	—	^∗∗∗^	^∗∗^	^∗∗∗^	^∗∗∗^	^∗∗∗^
Strain	—	^∗∗∗^	^∗∗∗^	^∗∗∗^	^∗∗∗^	^∗∗∗^
Diet × strain	—	^∗∗∗^	^∗∗∗^	^∗∗∗^	^∗∗∗^	^∗∗∗^

*Note:* D0.36, D0.96, D1.05, D1.15, D1.23 and D1.41 represent the six experimental diets formulated by including 0%, 0.67%, 0.77%, 0.87%, 0.97% and 1.17% methionine, respectively, in place of cellulose, resulting in analysed dietary methionine concentrations of 0.36%, 0.96%, 1.05%, 1.15%, 1.23% and 1.41%, respectively. Values given as mean ± SE. Values with different lowercase superscript letters within columns are significantly different (*p* < 0.05). Two‐way ANOVA:  ^∗∗^ *p* < 0.005,  ^∗∗∗^ *p* < 0.001.

### 3.3. Serum Antioxidant Capacity

There was a significant effect of dietary methionine, strain and their interaction on all serum antioxidant parameters (*p* < 0.05). As presented in Table 7, significantly higher (*p* < 0.05) levels of SOD (8.70 ± 0.19 U/mL) and GSH‐Px (37.00 ± 3.11 U/mL), and significantly lower (*p* < 0.05) levels of MDA (1.38 ± 0.02 nmol/mL) were recorded at an inclusion of methionine at 10.5 g/kg of the diet (D1.05) for the local strain. For the improved strain, significantly higher (*p* < 0.05) levels of SOD (15.76 ± 3.12 U/mL) and GSH‐Px (6.45 ± 0.12 U/mL), and significantly lower (*p* < 0.05) levels of MDA (5.35 ± 1.02 nmol/mL) were recorded at an inclusion of methionine at 11.5 g/kg of the diet (D1.15).

**Table 7:** Serum superoxide dismutase (SOD), glutathione peroxidase (GSH‐Px) and malondialdehyde (MDA) levels of the local and improved strains of Nile tilapia fed experimental diets containing graded levels of methionine for 84 days.

Diet	Strain	Parameters		
		SOD (U/mL)	GSH‐Px (U/mL)	MDA (nmol/mL)
D0.36	Local	6.70 ± 1.11^e^	27.33 ± 2.02^f^	2.38 ± 0.03^a^
D0.96	Local	7.17 ± 1.01^d^	30.67 ± 2.01^d^	2.05 ± 0.03^c^
D1.05	Local	8.70 ± 0.19^a^	37.00 ± 3.11^a^	1.38 ± 0.02^f^
D1.15	Local	7.57 ± 0.08^b^	32.67 ± 3.12^b^	1.78 ± 0.04^e^
D1.23	Local	7.27 ± 1.05^c^	31.67 ± 3.02^c^	1.92 ± 0.03^d^
D1.41	Local	6.80 ± 0.08^e^	28.67 ± 2.01^e^	2.23 ± 0.03^b^
D0.36	Improved	12.45 ± 3.19^e^	5.18 ± 1.08^d^	6.83 ± 1.02^b^
D0.96	Improved	13.75 ± 3.03^d^	5.75 ± 1.11^c^	6.65 ± 0.12^c^
D1.05	Improved	14.45 ± 4.11^b^	6.08 ± 1.01^b^	6.07 ± 1.00^d^
D1.15	Improved	15.76 ± 3.12^a^	6.45 ± 0.12^a^	5.35 ± 1.02^e^
D1.23	Improved	14.25 ± 3.08^c^	6.02 ± 1.08^b^	6.97 ± 0.11^a^
D1.41	Improved	12.38 ± 3.12^f^	5.08 ± 1.19^e^	6.83 ± 1.02^b^
Diet				
D0.36	—	9.57 ± 4.46^e^	16.25 ± 2.08^f^	4.60 ± 1.09^a^
D0.96	—	10.46 ± 3.60^d^	18.21 ± 2.85^d^	4.35 ± 1.12^d^
D1.05	—	11.57 ± 4.00^b^	21.54 ± 2.05^a^	3.72 ± 1.00^e^
D1.15	—	11.66 ± 4.01^a^	19.56 ± 2.74^b^	3.56 ± 1.08^f^
D1.23	—	10.76 ± 3.17^c^	18.84 ± 2.44^c^	4.44 ± 1.11^c^
D1.41	—	9.59 ± 4.56^e^	16.87 ± 2.24^e^	4.53 ± 0.90^b^
Strain				
Local	—	7.37 ± 3.11^b^	31.33 ± 3.00^a^	1.96 ± 0.05^b^
Improved	—	13.84 ± 4.08^a^	5.76 ± 1.56^b^	6.45 ± 2.34^a^
Two‐way ANOVA				
Diet	—	^∗∗∗^	^∗∗^	^∗∗∗^
Strain	—	^∗∗∗^	^∗∗∗^	^∗∗∗^
Diet × strain	—	^∗∗∗^	^∗∗∗^	^∗∗∗^

*Note:* D0.36, D0.96, D1.05, D1.15, D1.23 and D1.41 represent the six experimental diets formulated by including 0%, 0.67%, 0.77%, 0.87%, 0.97% and 1.17% methionine, respectively, in place of cellulose, resulting in analysed dietary methionine concentrations of 0.36%, 0.96%, 1.05%, 1.15%, 1.23% and 1.41%, respectively. Values given as mean ± SE. Values with different lowercase superscript letters within columns are significantly different (*p* < 0.05). Two‐way ANOVA:  ^∗∗^ *p* < 0.005,  ^∗∗∗^ *p* < 0.001.

### 3.4. Haematological Profile

There was a significant effect of dietary methionine, strain and their interaction (*p* < 0.05) on all the haematological parameters, including the derived parameters MCV, MCH and MCHC. As presented in Table 8, the local strain showed significantly higher (*p* < 0.05) levels of RBC (2.14 ± 0.45 10^6^/µL), Hct (30.10 ± 2.98%), Hgb (6.60 ± 0.19 g/dL), WBC (59.00 ± 5.96 10^3^/µL), platelets (382.00 ± 8.38 10^3^/µL), lymphocytes (53.80 ± 3.98 10^3^/µL) and monocytes (4.80 ± 0.02 10^3^/µL), and significantly lower (*p* < 0.05) levels of neutrophils (10.90 ± 1.73%) at 10.5 g/kg dietary inclusion of methionine (D1.05). The improved strain showed significantly higher (*p* < 0.05) levels of RBC (1.48 ± 0.35 10^6^/µL), Hct (24.70 ± 2.84%), Hgb (6.70 ± 0.53 g/dL), WBC (94.10 ± 5.18 10^3^/µL), platelets (386.00 ± 10.13 10^3^/µL), lymphocytes (49.30 ± 3.20 10^3^/µL) and monocytes (3.80 ± 0.58 10^3^/µL), and significantly lower (*p* < 0.05) levels of neutrophils (10.40 ± 2.83%) at 11.5 g/kg dietary inclusion of methionine (D1.15).

**Table 8:** Haematological profile of the local and improved strains of Nile tilapia fed experimental diets containing graded levels of methionine for 84 days.

Diet	Strain	Parameters										
		RBC (10^6^/µL)	Haematocrit (Hct; %)	Haemoglobin (Hgb; g/dL)	WBC (10^3^/µL)	Platelets (10^3^/µL)	Lymphocytes (10^3^/µL)	Monocytes (10^3^/µL)	Neutrophils (%)	MCV (fL)	MCH (pg)	MCHC (g/dL)
D0.36	Local	1.46 ± 0.91^e^	22.60 ± 1.04^f^	5.50 ± 0.39^e^	40.00 ± 4.85^f^	348.00 ± 9.02^f^	33.40 ± 3.38^f^	2.10 ± 0.03^e^	15.70 ± 1.73^a^	154.80 ± 8.00^a^	37.67 ± 3.82^a^	24.34 ± 2.83^a^
D0.96	Local	1.97 ± 0.48^c^	24.40 ± 2.43^d^	5.80 ± 0.37^d^	55.10 ± 5.46^c^	376.00 ± 9.59^d^	48.30 ± 3.10^d^	3.20 ± 0.34^c^	12.80 ± 1.13^d^	123.90 ± 9.48^f^	29.44 ± 2.44^f^	23.77 ± 2.58^b^
D1.05	Local	2.14 ± 0.45^a^	30.10 ± 2.98^a^	6.60 ± 0.19^a^	59.00 ± 5.96^a^	382.00 ± 8.38^a^	53.80 ± 3.98^a^	4.80 ± 0.02^a^	10.90 ± 1.73^e^	140.65 ± 8.56^c^	30.84 ± 2.04^d^	21.93 ± 2.57^f^
D1.15	Local	2.04 ± 0.12^b^	27.40 ± 2.00^b^	6.20 ± 0.48^b^	58.00 ± 5.84^b^	381.00 ± 10.49^b^	51.50 ± 3.36^b^	3.60 ± 0.84^b^	12.80 ± 1.49^d^	134.31 ± 8.09^e^	30.39 ± 3.96^e^	22.62 ± 2.29^d^
D1.23	Local	1.92 ± 0.16^c^	26.80 ± 2.53^c^	6.00 ± 0.02^c^	54.00 ± 4.56^d^	379.00 ± 12.28^c^	48.90 ± 3.02^c^	2.80 ± 0.84^d^	13.30 ± 1.80^c^	139.58 ± 9.04^d^	31.25 ± 2.68^c^	22.39 ± 2.35^e^
D1.41	Local	1.57 ± 0.38^d^	23.20 ± 2.59^e^	5.50 ± 0.28^e^	53.80 ± 4.76^e^	368.00 ± 11.28^e^	41.00 ± 3.19^e^	2.30 ± 0.82^e^	15.50 ± 1.01^b^	147.77 ± 7.83^b^	35.03 ± 2.55^b^	23.71 ± 2.04^c^
D0.36	Improved	1.30 ± 0.48^d^	21.30 ± 1.16^d^	5.30 ± 0.45^e^	80.30 ± 5.83^e^	342.00 ± 9.12^e^	46.10 ± 3.00^c^	1.30 ± 0.86^e^	14.60 ± 1.38^a^	163.85 ± 7.85^c^	40.77 ± 3.86^e^	24.88 ± 2.57^e^
D0.96	Improved	1.38 ± 0.93^c^	22.00 ± 1.11^bc^	6.10 ± 0.43^c^	88.90 ± 4.57^c^	344.00 ± 10.37^c^	41.70 ± 3.73^e^	2.80 ± 0.98^c^	12.50 ± 2.92^b^	159.42 ± 7.85^f^	44.20 ± 3.56^c^	27.73 ± 2.95^a^
D1.05	Improved	1.40 ± 0.37^b^	22.80 ± 1.45^b^	6.20 ± 0.64^b^	93.60 ± 5.06^a^	358.00 ± 9.93^b^	42.80 ± 3.27^d^	3.30 ± 0.95^b^	11.50 ± 1.28^c^	162.86 ± 8.57^d^	44.29 ± 3.45^b^	27.19 ± 2.76^b^
D1.15	Improved	1.48 ± 0.35^a^	24.70 ± 2.84^a^	6.70 ± 0.53^a^	94.10 ± 5.18^a^	386.00 ± 10.13^a^	49.30 ± 3.20^a^	3.80 ± 0.58^a^	10.40 ± 2.83^e^	166.89 ± 8.78^a^	45.27 ± 3.86^a^	27.13 ± 3.56^c^
D1.23	Improved	1.38 ± 0.92^c^	22.40 ± 2.47^b^	6.10 ± 0.57^c^	91.00 ± 5.74^b^	343.00 ± 11.20^d^	46.10 ± 3.26^c^	3.30 ± 0.02^b^	11.10 ± 1.73^d^	162.32 ± 9.57^e^	44.20 ± 3.58^c^	27.23 ± 2.63^b^
D1.41	Improved	1.31 ± 0.18^d^	21.80 ± 2.03^cd^	5.60 ± 0.94^d^	87.10 ± 5.65^d^	342.00 ± 10.29^e^	47.90 ± 3.82^b^	1.70 ± 0.29^d^	14.30 ± 1.45^a^	166.41 ± 8.76^b^	42.75 ± 3.34^d^	25.69 ± 2.94^d^
Diet												
D0.36	—	1.38 ± 0.84^d^	21.95 ± 2.85^e^	5.40 ± 0.28^e^	60.15 ± 4.75^e^	345.00 ± 11.28^e^	39.75 ± 3.27^e^	1.70 ± 0.92^f^	15.15 ± 1.00^a^	159.42 ± 9.06^a^	39.22 ± 2.57^a^	24.61 ± 2.38^d^
D0.96	—	1.68 ± 0.30^b^	23.20 ± 2.14^c^	5.95 ± 0.27^c^	72.00 ± 4.96^b^	360.00 ± 8.25^c^	45.00 ± 3.37^d^	3.00 ± 0.84^d^	12.65 ± 1.02^c^	141.66 ± 8.82^e^	36.82 ± 3.86^f^	25.75 ± 2.73^a^
D1.05	—	1.77 ± 0.38^a^	26.45 ± 2.54^a^	6.40 ± 0.03^a^	76.30 ± 5.46^a^	370.00 ± 9.93^b^	48.30 ± 3.28^b^	4.05 ± 0.92^a^	11.20 ± 1.30^f^	151.76 ± 8.47^c^	37.57 ± 3.45^e^	24.56 ± 2.48^d^
D1.15	—	1.76 ± 0.59^a^	26.05 ± 2.93^a^	6.45 ± 0.93^a^	76.05 ± 5.33^ab^	383.50 ± 12.26^a^	50.40 ± 3.83^a^	3.70 ± 0.20^b^	11.60 ± 1.83^f^	150.60 ± 8.45^d^	37.83 ± 2.85^c^	24.88 ± 2.84^b^
D1.23	—	1.65 ± 0.29^b^	24.6 ± 2.30^b^	6.05 ± 0.37^b^	72.50 ± 4.64^c^	361.00 ± 10.22^c^	47.50 ± 3.02^c^	3.05 ± 0.28^c^	12.20 ± 1.45^d^	150.95 ± 7.03^d^	37.73 ± 3.80^d^	24.81 ± 2.83^c^
D1.41	—	1.44 ± 0.50^c^	22.50 ± 2.38^d^	5.55 ± 0.83^d^	70.45 ± 5.18^d^	355.00 ± 10.19^d^	44.45 ± 3.92^e^	2.00 ± 0.39^e^	14.90 ± 1.27^b^	157.09 ± 7.82^b^	38.89 ± 2.57^b^	24.70 ± 2.09^e^
Strain												
Local	—	1.85 ± 0.84^a^	25.75 ± 2.37^a^	5.93 ± 0.37^b^	53.32 ± 5.28^b^	372.33 ± 9.12^a^	46.15 ± 3.28^b^	3.13 ± 0.96^a^	13.50 ± 1.28^a^	140.17 ± 7.43^b^	32.44 ± 2.46^b^	23.13 ± 2.29^b^
Improved	—	1.37 ± 0.36^b^	22.50 ± 2.58^b^	6.00 ± 0.03^a^	89.17 ± 5.72^a^	352.50 ± 10.25^b^	45.65 ± 3.87^a^	2.70 ± 0.38^b^	12.40 ± 1.82^b^	163.63 ± 7.92^a^	43.58 ± 3.90^a^	26.64 ± 2.28^a^
Two‐way ANOVA												
Diet (main effect)	—	^∗∗^	^∗∗^	^∗∗∗^	^∗∗^	^∗∗∗^	^∗∗∗^	^∗∗∗^	^∗∗∗^	^∗∗∗^	^∗∗∗^	^∗∗^
Strain (main effect)	—	^∗∗∗^	^∗∗∗^	^∗∗∗^	^∗∗∗^	^∗∗∗^	^∗∗∗^	^∗∗∗^	^∗∗∗^	^∗∗∗^	^∗∗∗^	^∗∗∗^
Interaction effect	—	^∗∗∗^	^∗∗∗^	^∗∗∗^	^∗∗∗^	^∗∗∗^	^∗∗∗^	^∗∗∗^	^∗∗∗^	^∗∗∗^	^∗∗∗^	^∗∗∗^

*Note:* D0.36, D0.96, D1.05, D1.15, D1.23 and D1.41 represent the six experimental diets formulated by including 0%, 0.67%, 0.77%, 0.87%, 0.97% and 1.17% methionine, respectively, in place of cellulose, resulting in analysed dietary methionine concentrations of 0.36%, 0.96%, 1.05%, 1.15%, 1.23% and 1.41%, respectively. Values given as mean ± SE. Values with different lowercase superscript letters within columns are significantly different (*p* < 0.05). Two‐way ANOVA:  ^∗∗^ *p* < 0.005,  ^∗∗∗^ *p* < 0.001.

### 3.5. Amino Acid Composition of the Fish Carcase

There was a significant effect of dietary methionine, strain, and their interaction on the amino acid composition of the fish carcase (*p* < 0.05), as shown in Table 9. An inclusion of methionine at 10.5 g/kg of the diet (D1.05) resulted in significantly higher (*p* < 0.05) levels of histidine (1.23% ± 0.01% DM), isoleucine (2.46% ± 0.01% DM), leucine (4.61% ± 0.02% DM), lysine (5.04% ± 0.02% DM), methionine (1.84% ± 0.01% DM), phenylalanine (2.58% ± 0.01% DM), threonine (2.52% ± 0.01% DM), tryptophan (0.68% ± 0.01% DM) and valine (2.77% ± 0.01% DM). For the improved strain, significantly higher (*p* < 0.05) levels of histidine (1.41% ± 0.01% DM), isoleucine (2.64% ± 0.01% DM), leucine (4.81% ± 0.03% DM), lysine (5.24% ± 0.03% DM), methionine (2.03% ± 0.01% DM), phenylalanine (2.77% ± 0.03% DM), threonine (2.71% ± 0.02% DM), tryptophan (0.85% ± 0.01% DM) and valine (2.95% ± 0.03% DM) in the fish carcase were recorded at an inclusion of methionine at 11.5 g/kg of the diet (D1.15).

**Table 9:** Carcase amino acid composition of the local and improved strains of Nile tilapia fed experimental diets containing graded levels of methionine for 84 days.

Diet	Strain	Amino acid composition (% DM)								
		Histidine	Isoleucine	Leucine	Lysine	Methionine	Phenylalanine	Threonine	Tryptophan	Valine
D0.36	Local	1.10 ± 0.01^f^	2.19 ± 0.02^e^	4.11 ± 0.04^f^	4.49 ± 0.04^d^	1.64 ± 0.01^f^	2.30 ± 0.02^f^	2.25 ± 0.02^d^	0.60 ± 0.01^c^	2.47 ± 0.02^f^
D0.96	Local	1.17 ± 0.01^c^	2.33 ± 0.02^c^	4.37 ± 0.04^c^	4.78 ± 0.05^c^	1.75 ± 0.02^c^	2.45 ± 0.02^c^	2.39 ± 0.02^c^	0.64 ± 0.01^b^	2.62 ± 0.03^c^
D1.05	Local	1.23 ± 0.01^a^	2.46 ± 0.02^a^	4.61 ± 0.02^a^	5.04 ± 0.02^a^	1.84 ± 0.01^a^	2.58 ± 0.01^a^	2.52 ± 0.01^a^	0.68 ± 0.01^a^	2.77 ± 0.01^a^
D1.15	Local	1.21 ± 0.01^b^	2.42 ± 0.02^b^	4.55 ± 0.04^b^	4.97 ± 0.05^b^	1.82 ± 0.02^b^	2.55 ± 0.03^b^	2.49 ± 0.02^b^	0.67 ± 0.01^a^	2.73 ± 0.03^b^
D1.23	Local	1.15 ± 0.01^d^	2.31 ± 0.02^c^	4.32 ± 0.04^d^	4.73 ± 0.04^c^	1.73 ± 0.01^d^	2.42 ± 0.02^d^	2.36 ± 0.02^c^	0.63 ± 0.01^b^	2.59 ± 0.02^d^
D1.41	Local	1.12 ± 0.01^e^	2.23 ± 0.02^d^	4.19 ± 0.04^e^	4.58 ± 0.04^c^	1.68 ± 0.01^e^	2.35 ± 0.02^e^	2.29 ± 0.02^d^	0.61 ± 0.01^c^	2.51 ± 0.02^e^
D0.36	Improved	1.27 ± 0.01^d^	2.37 ± 0.01^f^	4.30 ± 0.02^e^	4.69 ± 0.02^e^	1.82 ± 0.01^e^	2.48 ± 0.01^f^	2.43 ± 0.01^d^	0.77 ± 0.01^c^	2.65 ± 0.01^e^
D0.96	Improved	1.34 ± 0.01^c^	2.52 ± 0.02^c^	4.57 ± 0.03^c^	4.98 ± 0.03^c^	1.93 ± 0.01^c^	2.63 ± 0.02^c^	2.57 ± 0.02^c^	0.81 ± 0.01^b^	2.61 ± 0.02^f^
D1.05	Improved	1.39 ± 0.01^b^	2.61 ± 0.02^b^	4.74 ± 0.03^b^	5.17 ± 0.04^b^	2.00 ± 0.01^b^	2.73 ± 0.02^b^	2.67 ± 0.02^b^	0.84 ± 0.01^a^	2.91 ± 0.02^b^
D1.15	Improved	1.41 ± 0.01^a^	2.64 ± 0.01^a^	4.81 ± 0.03^a^	5.24 ± 0.03^a^	2.03 ± 0.01^a^	2.77 ± 0.02^a^	2.71 ± 0.02^a^	0.85 ± 0.01^a^	2.95 ± 0.02^a^
D1.23	Improved	1.33 ± 0.01^c^	2.49 ± 0.01^d^	4.52 ± 0.02^c^	4.92 ± 0.03^c^	1.91 ± 0.01^c^	2.60 ± 0.01^d^	2.54 ± 0.01^c^	0.80 ± 0.01^b^	2.78 ± 0.01^c^
D1.41	Improved	1.29 ± 0.01^d^	2.41 ± 0.01^e^	4.38 ± 0.03^d^	4.77 ± 0.03^d^	1.85 ± 0.01^d^	2.52 ± 0.01^e^	2.47 ± 0.01^d^	0.76 ± 0.01^c^	2.69 ± 0.01^d^
Diet	—	—	—	—	—	—	—	—	—	—
D0.36	—	1.18 ± 0.01^c^	2.28 ± 0.01^d^	4.20 ± 0.03^d^	4.59 ± 0.04^d^	1.73 ± 0.01^d^	2.39 ± 0.02^e^	2.34 ± 0.01^d^	0.68 ± 0.01^c^	2.56 ± 0.02^d^
D0.96	—	1.25 ± 0.01^b^	2.42 ± 0.01^b^	4.47 ± 0.03^b^	4.88 ± 0.03^b^	1.84 ± 0.01^b^	2.54 ± 0.02^b^	2.48 ± 0.01^b^	0.72 ± 0.01^b^	2.61 ± 0.02^c^
D1.05	—	1.31 ± 0.01^a^	2.53 ± 0.02^a^	4.67 ± 0.03^a^	5.10 ± 0.04^a^	1.92 ± 0.01^a^	2.65 ± 0.01^a^	2.59 ± 0.02^a^	0.76 ± 0.01^a^	2.84 ± 0.01^a^
D1.15	—	1.31 ± 0.01^a^	2.53 ± 0.02^a^	4.68 ± 0.02^a^	5.10 ± 0.04^a^	1.92 ± 0.01^a^	2.66 ± 0.01^a^	2.60 ± 0.01^a^	0.76 ± 0.01^a^	2.84 ± 0.01^a^
D1.23	—	1.24 ± 0.01^b^	2.40 ± 0.01^b^	4.42 ± 0.04^b^	4.82 ± 0.02^b^	1.82 ± 0.01^b^	2.51 ± 0.02^c^	2.45 ± 0.02^c^	0.71 ± 0.01^b^	2.68 ± 0.01^b^
D1.41	—	1.20 ± 0.01^c^	2.32 ± 0.01^c^	4.28 ± 0.03^c^	4.67 ± 0.03^c^	1.76 ± 0.01^c^	2.43 ± 0.01^d^	2.38 ± 0.01^d^	0.68 ± 0.01^c^	2.60 ± 0.02^c^
Strain										
Local	—	1.16 ± 0.01^b^	2.32 ± 0.01^b^	4.36 ± 0.04^b^	4.76 ± 0.03^b^	1.74 ± 0.01^b^	2.44 ± 0.02^b^	2.38 ± 0.01^b^	0.64 ± 0.01^b^	2.61 ± 0.01^b^
Improved	—	1.34 ± 0.01^a^	2.51 ± 0.01^a^	4.55 ± 0.03^a^	4.96 ± 0.04^a^	1.92 ± 0.01^a^	2.62 ± 0.01^a^	2.56 ± 0.01^a^	0.81 ± 0.01^a^	2.76 ± 0.02^a^
Two‐way ANOVA										
Diet	—	^∗∗∗^	^∗∗∗^	^∗∗∗^	^∗∗∗^	^∗∗∗^	^∗∗∗^	^∗∗∗^	^∗∗^	^∗∗∗^
Strain	—	^∗∗∗^	^∗∗∗^	^∗∗∗^	^∗∗∗^	^∗∗∗^	^∗∗∗^	^∗∗∗^	^∗∗∗^	^∗∗∗^
Diet × strain	—	^∗∗∗^	^∗∗∗^	^∗∗∗^	^∗∗∗^	^∗∗∗^	^∗∗∗^	^∗∗∗^	^∗∗^	^∗∗∗^

*Note:* D0.36, D0.96, D1.05, D1.15, D1.23 and D1.41 represent the six experimental diets formulated by including 0%, 0.67%, 0.77%, 0.87%, 0.97% and 1.17% methionine, respectively, in place of cellulose, resulting in analysed dietary methionine concentrations of 0.36%, 0.96%, 1.05%, 1.15%, 1.23% and 1.41%, respectively. Values given as mean ± SE. Values with different lowercase superscript letters within columns are significantly different (*p* < 0.05). Two‐way ANOVA:  ^∗∗^ *p* < 0.005,  ^∗∗∗^ *p* < 0.001.

### 3.6. Quadratic Regression Results

The quadratic regression model estimated the optimal dietary methionine requirement for the local strain at 0.908% for weight gain, 0.909% for SGR, and 0.964% for FCR (Figure 2). The model showed a high coefficient of determination for weight gain (*R* ^2^ = 0.733, *β*_1_ = 67.823, *β*_2_ = −37.323, *p* < 0.001), SGR (*R* ^2^ = 0.786, *β*_1_ = 1.657, *β*_2_ = −0.911, *p* < 0.001) and FCR (*R* ^2^ = 0.733, *β*_1_ = −1.153, *β*_2_ = 0.598, *p* < 0.001).

**Figure 2:**
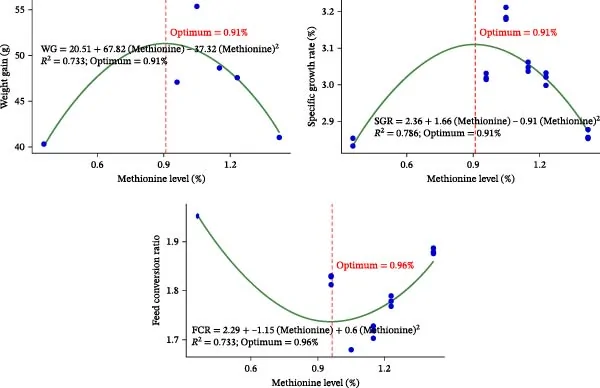
Quadratic regression results of the dietary methionine requirement of the local strain of Nile tilapia fed experimental diets containing graded levels of methionine for 84 days.

The fitted quadratic equations for the local strain were as follows:

Weight gain=20.51167.82337.323+methionine−methionine2.

SGR=2.35641.65720.9111+methionine−methionine2.

FCR=2.29201.15250.5976−methionine+methionine2.

The quadratic regression model estimated the optimal dietary methionine requirement for the improved strain at 0.913% for weight gain, 0.912% for SGR and 0.955% for FCR (Figure 3). The model showed a high coefficient of determination for weight gain (*R* ^2^ = 0.632, *β*_1_ = 45.832, *β*_2_ = –25.097, *p* < 0.001), SGR (*R* ^2^ = 0.645, *β*_1_ = 0.971, *β*_2_ = −0.532, *p* < 0.001) and FCR (*R* ^2^ = 0.799, *β*_1_ = −1.342, *β*_2_ = 0.702, *p* < 0.001).

**Figure 3:**
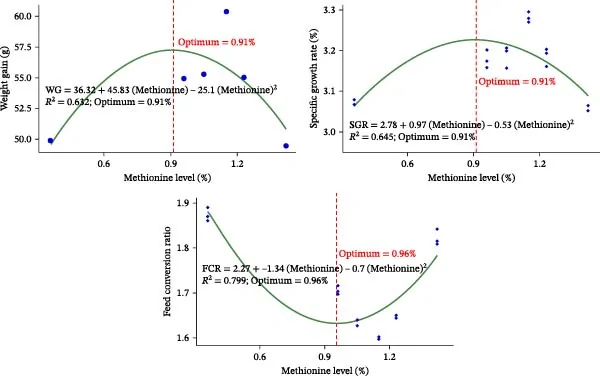
Quadratic regression results of the dietary methionine requirement of the improved strain of Nile tilapia fed experimental diets containing graded levels of methionine for 84 days.

The fitted quadratic equations for the improved strain were as follows:

Weight gain=36.32345.83225.097+methionine−methionine2.

SGR=2.78370.97080.5321+methionine−methionine2.

FCR=2.27371.34160.7023−methionine+methionine2.

## 4. Discussion

Methionine is one of the most limiting essential amino acids in feeds rich in plant or insect protein meals [11, 12]. While insect meals, such as BSFL, provide a favourable amino acid profile, they are still relatively low in essential sulphur‐containing amino acids, especially methionine, when compared to fishmeal [12]. Methionine serves as a primary sulphur and methyl group donor for the biosynthesis of important metabolites, including cysteine and taurine [15].

Both strains exhibited optimal performance at intermediate dietary methionine inclusion levels, with the improved strain attaining higher performance at a slightly higher methionine inclusion level. The pattern of improved performance at intermediate methionine inclusion levels is consistent with the classical response observed in dietary methionine studies in *O. niloticus* [3, 18, 19, 41] and other fish species, such as silver pompano, *Trachinotus blochii* [16], barramundi, *Lates calcarifer* [42] and African catfish, *Clarias gariepinus* [43]. Deficiency leads to reduced performance, while oversupply can result in amino acid antagonism, increased deamination, and overall reduced performance [28, 44, 45]. The observed improvements in weight gain, FCR, PER and somatic indices at optimal methionine levels indicate enhanced protein utilisation and reduced metabolic wastage. Methionine plays a crucial role in the initiation of protein synthesis, methyl‐group donation (through S‐adenosylmethionine), and trans‐sulphuration pathways involved in the synthesis of cysteine, taurine and glutathione [45–48]. Therefore, adequate availability of methionine enhances muscle protein deposition, reduces amino acid catabolism for energy, promotes lipid and energy metabolism and improves overall nitrogen retention and growth in fish [18, 49–51].

Serum biochemical and antioxidant indices offer detailed insights into metabolic function, organ health, immune competence and nutritional status of fish. Serum biochemistry integrates multiple physiological processes, making it one of the most informative tools for assessing dietary impacts in fish nutrition [19, 52]. In the present study, the higher HDL concentration together with lower triglycerides, LDL and FFAs at intermediate methionine inclusion levels may therefore indicate a more favourable regulation of lipid transport and turnover. HDL is involved in the transport of lipids, including the redistribution of cholesterol, whereas triglycerides and FFAs reflect circulating lipid availability and mobilisation. The observed pattern is consistent with the possibility that an adequate methionine supply supported balanced lipid metabolism, whereas insufficient or excessive methionine may disrupt specific lipid metabolic pathways, as reported by Zhou et al. [53] and Song et al. [54]. Zhou et al. [53] reported that methionine restriction increased *de novo* lipogenesis, lipolysis and fatty acid oxidation, resulting in reduced fat accumulation, indicating that methionine availability can alter the balance among lipid synthesis, mobilisation and oxidation. Similarly, Song et al. [54] demonstrated that both methionine deficiency and excess altered lipid metabolism in *Pelteobagrus fulvidraco* but through different pathways. Methionine deficiency reduced lipolysis and lipid transport, whereas methionine excess reduced lipid oxidation and transport and increased lipogenesis. These findings suggest that an appropriate dietary methionine supply may be important for maintaining a balanced regulation of lipid synthesis, mobilisation, transport and oxidation.

The lower glucose and cortisol concentrations observed at the intermediate methionine levels may further indicate reduced metabolic and stress‐related burden [55, 56]. Additionally, decreased BUN, AST and ALT may indicate more efficient nitrogen utilisation and reduced hepatic or tissue damage [57, 58]. Moreover, the higher activities of SOD and GSH‐Px indicate enhanced antioxidant defense capacity as these enzymes are key components of enzymatic protection against reactive oxygen species (ROS), while lower MDA concentrations indicate reduced lipid peroxidation and oxidative damage to cellular membranes [59].

Haematological markers provide broad insights into the systemic physiological state of fish. These parameters reflect the oxygen‐carrying capacity, immunity, and health status of fish [16, 60, 61]. Dietary quality and ingredient composition significantly influence haematology [52, 62]. RBCs, Hgb and Hct increase the blood’s capacity to transport oxygen to tissues, supporting more efficient cellular respiration and energy production [63, 64], while WBC enhances innate and adaptive immune responses [65, 66]. The higher RBC, Hgb, Hct and WBC observed at intermediate methionine inclusion levels in the present study indicate improved oxygen transport efficiency, better immune response and good nutritional status of the fish. However, since increased leukocyte counts can also be associated with physiological stress or pathogen exposure [67, 68], further immunological assessments (e.g., serum enzyme activities, which have been analysed in the present study) are necessary for confirming immune enhancement.

Significantly higher concentrations of essential amino acids were observed at dietary methionine inclusion of 10.5 g/kg in the local strain and 11.5 g/kg in the improved strain. Adequate dietary methionine facilitates more efficient utilisation of other amino acids, which are no longer constrained by methionine deficiency, thereby supporting coordinated protein synthesis and promoting the incorporation of absorbed amino acids into muscle protein [69, 70]. These findings also have implications for fillet quality as the higher concentrations of methionine and other essential amino acids at the optimal methionine inclusion levels suggest that appropriate dietary methionine supply can improve not only growth performance but also the amino acid profile and nutritional value of the resulting fish fillet. Therefore, optimising dietary methionine may contribute to the production of tilapia with enhanced protein quality and greater nutritional value for consumers.

Dietary cysteine can reduce the amount of methionine required to satisfy the total sulphur amino acid (TSAA) requirements of tilapia [13, 71]. Previous studies have reported that cysteine may replace dietary methionine up to 49% [71] and 47% [13] in *O. niloticus* and up to 40.3% in *Catla catla* [14], 40% in rohu, *Labeo rohita* [72] and 40.4%–49.2% in yellowtail kingfish, *Seriola lalandi* [15]. In the current study, a constant cysteine level of 4.2 g/kg of feed was used, corresponding to 30% of the highest level of dietary methionine used and 43%–46% of the estimated optimum dietary methionine level. The regression analyses estimated the optimum dietary methionine requirements of *O. niloticus* fed BSFL‐containing diets at 0.908%–0.964% (9.08–9.64 g/kg) of the diet for the local strain and at 0.913%–0.955% (9.13–9.55 g/kg) of the diet for the improved strain, with a cysteine level of 4.2 g/kg. The optimal dietary methionine requirement levels for *O. niloticus* in the present study were higher than the levels reported by Yossa et al. [21] that reported 7.5–8.0 g/kg of diet with a cysteine level of 5.0 g/kg of diet, Michelato et al. [41] that reported 9.0–9.9 g of methionine + cysteine per kg of diet and [71] that reported 4.9 g/kg of diet or 17.5 of dietary protein with cysteine level of 4.5 g/kg of diet. However, the optimal levels found in the present study are lower than the levels reported by Xiang et al. [18] that reported 11.3–11.6 g/kg of diet (35.2–36.1 g/kg of dietary protein) with a cysteine level at 3.0 g/kg of diet and Garg et al. [20] that reported 12.1–14.1 g/kg of diet with a cysteine level of 3.2 g/kg of diet. Nevertheless, the optimal methionine requirement levels found in our study are in tandem with He et al. [19], who reported an optimal level of 9.12 g/kg of diet with a cysteine level of 0.83 g/kg of diet. These differences could be attributed to the variations in dietary cysteine levels in the studies. Accordingly, Nguyen and Davis [71] and Yossa et al. [21] used higher cysteine levels, reporting lower methionine requirements, while Xiang et al. [18] and Garg et al. [20] used lower cysteine levels, reporting higher methionine requirements. Moreover, BSFL differs from fish meal and soybean meal in amino acid digestibility, chitin content, and nutrient availability, potentially altering sulphur amino acid utilisation [11, 12], explaining the differences between the current study and the previous studies.

The optimal dietary methionine requirements estimated by the regression analyses remained nearly equal for both strains, despite the improved strain showing numerically better values for all the analysed parameters. This may indicate an improved capacity for amino acid utilisation in genetically improved tilapia lines. Selectively bred strains, such as the GIFT and Sagana strains, are known to exhibit superior growth, nutrient assimilation ability, enhanced metabolism, improved feed efficiency and immune response compared to unimproved or wild‐type strains [22–25]. These enhanced physiological traits may allow improved strains to utilise amino acid levels more efficiently than local strains. Moreover, the significant effects of dietary methionine, strain, and their interaction on the analysed parameters emphasise the importance of aligning dietary formulations not only with species‐specific requirements but also with strain‐specific nutritional needs. Similar comparative effects between diet and genotype have been reported by Genschick et al. [26], Fatan et al. [27], and Yossa et al. [21], who showed that different tilapia strains respond differently to identical diets.

## 5. Conclusions

The present study shows that the optimum dietary methionine requirement of both the local and improved strains fed BSFL‐containing diets ranges from 9.1–9.6 g/kg of diet or 26–27 g/kg of dietary protein, with a cysteine level of 4.2 g/kg. Regression analysis showed that both strains have nearly identical optimal dietary methionine requirements on weight gain, SGR and FCR. Moreover, significant effects of dietary methionine, strain and their interaction on the analysed parameters were observed, underscoring the need to tailor dietary formulations not only to species‐specific requirements but also to strain‐specific nutritional needs for optimal biological performance and aquaculture productivity. Another important implication of the study is the confirmation that BSFL‐containing diets, when properly balanced with essential amino acids such as methionine, can effectively support optimal growth and physiological performance in *O. niloticus*. Insect meals offer high protein content, a favourable lipid profile and a low environmental footprint, and the success of their inclusion in fish diets depends heavily on amino acid optimisation strategies.

## Funding

This work was supported by the following organisations and agencies: the Norwegian Agency for Development Cooperation (NORAD) (Grant SAFA‐21/0004); Australian Centre for International Agricultural Research (ACIAR) (ProteinAfrica—Grant LS/2020/154); Global Affairs Canada (BRAINS project: P011585); European Commission (HORIZON 101060762 NESTLER and HORIZON 101136739 INNOECOFOOD); Bill and Melinda Gates Foundation (INV‐032416); the Curt Bergfors Foundation Food Planet Prize Award; the Swedish International Development Cooperation Agency (Sida); the Swiss Agency for Development and Cooperation (SDC); the Australian Centre for International Agricultural Research (ACIAR); the Government of Norway; the German Federal Ministry for Economic Cooperation and Development (BMZ); and the Government of the Republic of Kenya.

## Disclosure

The views expressed herein do not necessarily reflect the official opinion of the donors.

## Conflicts of Interest

The authors declare no conflicts of interest.

## Data Availability

The data that support the findings of this study are available from the corresponding author upon reasonable request.
